# Small Intestinal Bacterial Overgrowth in Patients with Irritable Bowel Syndrome: Clinical Characteristics, Psychological Factors, and Peripheral Cytokines

**DOI:** 10.1155/2016/3230859

**Published:** 2016-06-09

**Authors:** Hua Chu, Mark Fox, Xia Zheng, Yanyong Deng, Yanqin Long, Zhihui Huang, Lijun Du, Fei Xu, Ning Dai

**Affiliations:** ^1^Department of Gastroenterology, Sir Run Run Shaw Hospital, School of Medicine, Zhejiang University, Hangzhou 310016, China; ^2^Division of Gastroenterology & Hepatology, University Hospital Zürich, Zürich, Switzerland; ^3^Zürich Centre for Integrative Human Physiology (ZIHP), University of Zürich, Zürich, Switzerland

## Abstract

Small intestinal bacterial overgrowth (SIBO) has been implicated in the pathogenesis of irritable bowel syndrome (IBS). Psychosocial factors and low-grade colonic mucosal immune activation have been suggested to play important roles in the pathophysiology of IBS. In total, 94 patients with IBS and 13 healthy volunteers underwent a 10 g lactulose hydrogen breath test (HBT) with concurrent ^99m^Tc scintigraphy. All participants also completed a face-to-face questionnaire survey, including the Hospital Anxiety and Depression Scale, Life Event Stress (LES), and general information. Serum tumour necrosis factor-*α*, interleukin- (IL-) 6, IL-8, and IL-10 levels were measured. The 89 enrolled patients with IBS and 13 healthy controls had no differences in baseline characteristics. The prevalence of SIBO in patients with IBS was higher than that in healthy controls (39% versus 8%, resp.; *p* = 0.026). Patients with IBS had higher anxiety, depression, and LES scores, but anxiety, depression, and LES scores were similar between the SIBO-positive and SIBO-negative groups. Psychological disorders were not associated with SIBO in patients with IBS. The serum IL-10 level was significantly lower in SIBO-positive than SIBO-negative patients with IBS.

## 1. Introduction

Irritable bowel syndrome (IBS) is a common functional gastrointestinal disorder (FGID) characterised by recurrent abdominal pain or discomfort associated with alterations in stool frequency and/or consistency, for which there is no apparent physical or biochemical cause to explain the symptoms. About 10–20% [1] of adults in western countries and 5–10% [2–4] of Asian adults are affected by IBS. It seriously affects quality of life and consumes vast medical resources. The pathogenesis and pathophysiology of IBS are complex and remain incompletely understood. Visceral hypersensitivity and abnormal gastrointestinal motility are possible major pathophysiologies, but other factors may include brain-gut dysfunction, psychological disorders, dietary issues, gastrointestinal infections, and genetics.

Small intestinal bacterial overgrowth (SIBO) has received increasing attention, and many studies have shown that the frequency of SIBO in patients with IBS is higher than that in a healthy population [5–8]. SIBO is a condition in which the small bowel is colonised by colonic bacteria, resulting in symptoms ranging from bloating and diarrhoea to weight loss and nutritional deficiencies. However, the diagnostic tests for SIBO remain controversial. Direct aspiration and culture of organisms are the gold standard for diagnosing SIBO, formally defined as the presence of >10^5^ colony-forming units in jejunal aspirate; however, this approach is obviously invasive and difficult to perform, and aspirates from the proximal jejunum lack sensitivity in all but the most severe cases of enteric dysfunction. The lactulose hydrogen breath test (LHBT) is the most commonly used method to diagnose SIBO, but its accuracy has been questioned by many scholars because of individual variations in small intestinal transit [9]. We published a paper confirming that LHBT alone is not a valid test for SIBO [10]; however, LHBT combined with scintigraphic measurements of the orocaecal transit time (OCTT) provides an accurate and reproducible diagnostic test for SIBO. In the present study, we used this method to diagnose SIBO.

The main mechanisms of the development of SIBO are thought to include structural changes in the gastrointestinal tract, disordered gastric and/or small intestinal peristalsis, and disruption of normal mucosal small intestinal defences [11]. Abnormally low intestinal motility and immune activation may explain SIBO in a patient with IBS. Bacterial products, such as endotoxins, can affect gut motility [12]. Gut bacteria are also important for activating an immune response. Immune-mediated cytokines have multiple actions, including altered epithelial secretion, exaggerated nociceptive signalling, and abnormal motility [13]. Together, these changes may lead to IBS.

There is also considerable evidence that psychological and social influences can affect the perception of symptoms, healthcare-seeking behaviours, and outcomes in patients with FGIDs, particularly those with IBS [14, 15]. Little information is available about anxiety or depression in patients with IBS and SIBO. However, there is a large body of work demonstrating that patients with IBS have low-grade immune activation, and associations between psychological state and stress and immune activation have been detected in mucosa [16–19]. Results from animal experiments suggest that low-grade gut inflammation can alter gastrointestinal tract motor function [20] and that gut motility abnormalities can further predispose to bacterial overgrowth.

However, the relationships among SIBO, psychological factors, and proinflammatory and anti-inflammatory cytokines have not been established in patients with IBS. Thus, this study was designed to examine the clinical characteristics, psychological states, and serum cytokine levels in patients with IBS and SIBO to better understand the pathology of IBS.

## 2. Materials and Methods

The study was approved by the Ethics Committee of Sir Run Run Shaw Hospital (reference number, 20070823-2). All participants provided written informed consent before the study.

### 2.1. Participants

Data from 94 consecutive patients who met the Rome III criteria for IBS and 13 healthy volunteers with no history of gastrointestinal symptoms were studied. Patients had no alarm symptoms and no evidence of relevant organic diseases on colonoscopy, routine blood tests, or faecal microbiology. The participants were from the same population as those used in a previous study [10].

### 2.2. Assessment of Psychosocial Status and General Information

Psychological status was assessed with the Hospital Anxiety and Depression Score (HADS), and psychosocial stress was assessed by the Life Event Stress (LES) scale of Miller and Rahe, as modified and validated for use in Chinese populations [21]. A HADS score of ≥11 was considered to represent clinically significant anxiety or depression, with a cut-off score of ≥8 for diagnosing borderline neurosis. Anxiety and depression were defined as scores of ≥8 on each respective scale for the categorical analysis. The subject's demographic data (age, sex, marital status, education, profession, and income range), smoking, alcohol consumption, and medical and surgical history were recorded.

### 2.3. Combined LHBT and Scintigraphic Orocaecal Transit (SOCT) Study

The subjects were instructed to avoid foods containing incompletely absorbed carbohydrates, such as bread, corn, pasta, and potatoes, on the evening before the breath test and then underwent a minimum 12 h fast to minimise basal hydrogen excretion [22]. Immediately before the procedure, the subjects used 30 mL of antiseptic mouthwash (1.5% compound borax solution; Winguidehp, Shanghai, China) to eliminate lactulose fermentation due to oropharyngeal bacteria. Other extraintestinal influences on breath hydrogen concentrations, such as cigarette smoke, physical exercise, and hyperventilation [23], were avoided during the test. Subjects fasted for the duration of the test.

After determining the baseline H_2_ breath concentration, all subjects underwent combined LHBT/scintigraphy. As described previously [24], 10 g of lactulose (15 mL; Duphalac Solvay Pharmaceuticals B.V., Weesp, The Netherlands) labelled with 37 MBq ^99m^Tc-diethylene triamine pentaacetic acid (HTA Co. Ltd., Beijing, China) was ingested with 100 mL of water. The subjects were placed in the supine position with a gamma camera (Millennium VG; General Electric, Milwaukee, WI, USA) monitoring the abdomen. End-expiratory breath samples were collected concurrently with scintigraphic images after the meal and then every 15 min for up to 3 h using a portable analyser with a sensitivity of ±1 ppm (Micro H_2_ Meter; Micro Medical Limited, Chatham, UK). The geometric mean of the anterior and posterior values was used for scintigraphy to correct for depth changes (geometric mean counts = square root [anterior counts × posterior counts]) corrected for radioisotope decay [25]. The scintigraphy images were reviewed independently and in a blinded manner by two investigators to determine the arrival of the tracer in the caecal region of interest. The OCTT was defined as the time at which at least 5% of the administered isotope dose had accumulated in the caecal region [9, 24]. The temporal association between increased breath H_2_ and arrival of the marker in the caecum was assessed.

### 2.4. Diagnostic Criteria

**Figure 1:**
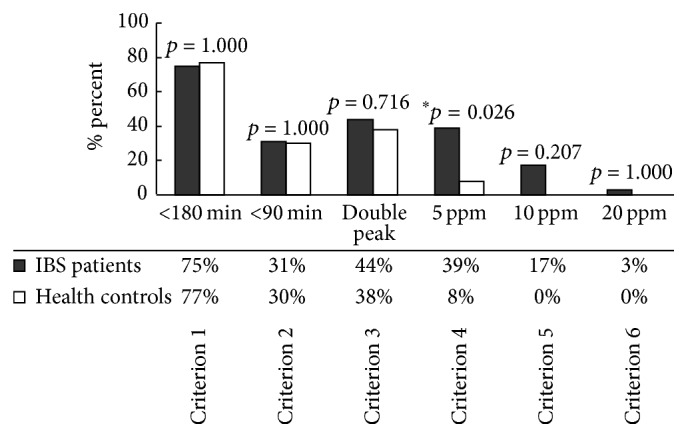
SIBO prevalence in IBS patients and healthy controls as determined by six published diagnostic criteria. Only Criterion 4 for the combined lactulose HBT/SOCT indicated a higher prevalence of SIBO in patients than controls (which has been published). Criterion 1: a H_2_ rise of ≥20 ppm within 180 min; Criterion 2: a H_2_ rise of ≥20 ppm within 90 min; Criterion 3: dual breath H_2_ peaks, a 12 ppm increase in breath H_2_ over baseline with a decrease of ≥5 ppm before the second peak; Criterion 4: initial H_2_ rise, involving at least two consecutive values ≥5 ppm above baseline, commenced at least 15 min before an increase of radioactivity (≥5% of administered dose) in the caecal region; Criterion 5: initial H_2_ rise, involving at least two consecutive values ≥10 ppm above baseline, commenced at least 15 min before an increase of radioactivity (≥5% of administered dose) in the caecal region; Criterion 6: initial H_2_ rise, involving at least two consecutive values ≥20 ppm above baseline, commenced at least 15 min before an increase of radioactivity (≥5% of administered dose) in the caecal region; ^*∗*^ *p* < 0.05.

The diagnostic criterion for SIBO was an initial H_2_ increase involving at least two consecutive values of ≥5 ppm above baseline, beginning at least 15 min before an increase in radioactivity (≥5% of administered dose) in the caecal region. In previous publication [10], we have demonstrated that combined LHBT/SOCT is a valid method for noninvasive diagnosis of SIBO. A 5 ppm increase in breath H_2_ prior to the arrival of cecal contrast may identify a subset of IBS patients that have good clinical outcomes following antibiotic therapy.  Figure 1 showed the results that had been published.

### 2.5. Serum Cytokine Tests

Twelve-hour fasting venous blood samples were drawn from patients. Whole-blood samples (2 mL) were collected in tubes containing ethylenediaminetetraacetic acid. The samples were centrifuged immediately, and serum was frozen at −80°C until further use. Serum tumour necrosis factor- (TNF-) *α*, interleukin- (IL-) 6, IL-8, and IL-10 levels were quantified using enzyme-linked immunosorbent assay kits (eBioscience, San Diego, CA, USA) according to the manufacturer's protocols. Optical density was measured at a wavelength of 450 nm. Density values were correlated linearly with the concentrations of cytokine standards.

### 2.6. Statistical Analysis

All statistical analyses were performed using SPSS version 19.0 for Windows software (SPSS Inc., Chicago, IL, USA). All variables are expressed as means ± standard deviations or medians with quartiles, as appropriate. Student's *t*-test was used to compare means, and the Mann-Whitney *U* test was used as a nonparametric statistical test. We used the *χ* ^2^ test for qualitative data comparisons. Alpha values of <0.05 were considered statistically significant.

## 3. Results

### 3.1. Study Population

In total, 94 patients with IBS and 13 healthy volunteers with no history of gastrointestinal symptoms were analysed. Five patients were excluded (*n* = 4 hydrogen nonproducers, *n* = 1 pancreatic cancer diagnosed after study entry). The patients and healthy controls were similar in age and sex (Table 1). Among the 89 subjects, most of the patients with IBS had diarrhoea-predominant IBS (IBS-D; 70.8%) or mixed type IBS (IBS-M; 23.6%); very few had constipation-predominant IBS (IBS-C; 3.4%) or the unsubtyped types (IBS-U; 2.2%) (Table 2).

According to the diagnostic criterion, 35/89 (39%) patients and 1/13 (8%) were diagnosed as SIBO-positive, whereas the others were SIBO-negative. The prevalence of SIBO in patients with IBS was higher than that in healthy controls (*p* = 0.026), as we showed previously. The demographic and clinical characteristics were similar in patients with IBS with and without SIBO, except marital status (Table 3).  Figure 1 had showed the reason that we selected these criteria and the paper had been published in the Journal of Neurogastroenterology and Motility [10].

### 3.2. Psychological State Reflected by HADS and Life Event Stress (LES)

Patients with IBS had higher anxiety, depression, and LES scores than did healthy controls (Table 1). However, the SIBO-positive and SIBO-negative patients exhibited similar scores for anxiety (5.37 ± 3.61 versus 5.04 ± 2.85, resp.; *p* = 0.628) and depression (5.86 ± 3.90 versus 5.30 ± 2.87, resp.; *p* = 0.437) (Table 4). Similarly, LES scores of patients with IBS and SIBO were comparable to those of patients without SIBO.

### 3.3. Serum Cytokines

The IL-10 level was significantly lower in SIBO-positive than SIBO-negative patients with IBS (mean, 12.92 pg/mL [range, 11.40–14.85 pg/mL], versus mean, 14.03 pg/mL [range, 12.66–16.33 pg/mL], resp.; *p* = 0.026). No differences were observed in TNF-*α*, IL-6, or IL-8 levels between the groups (Table 5).

## 4. Discussion

SIBO has been suggested to play a role in the pathophysiology of IBS, but the reported prevalence of SIBO in patients with IBS varies widely depending on the geographical origin of the study population, methods for detection, and criteria for diagnosing SIBO. Our results demonstrate that the prevalence of SIBO was higher in patients with IBS than in healthy controls, as we showed previously [10]. The diagnostic criterion for SIBO by combining LHBT with scintigraphic measurement is certificated in our previous study [10]. We found no relationship between SIBO and age, sex, BMI, or alcohol consumption. Many studies have demonstrated that older age and female sex are predictors of SIBO [26–29]. More females than males are diagnosed with IBS, and SIBO is more common in older individuals, likely due to reduced intestinal motility with advancing age [29]. However, consistent with Rana's report, we found no relationship between SIBO and old age or sex [30]. In a retrospective review, Gabbard et al. [31] reported that moderate alcohol consumption is a strong risk factor for SIBO. Previous studies [32, 33] have demonstrated that alcoholics have higher rates of SIBO. The main mechanisms for the development of SIBO include structural changes in the gastrointestinal tract, disordered gastric and/or small intestinal peristalsis, and disruption of the normal mucosal defences of the small intestine [11]. However, we found no association between SIBO and alcohol consumption in the present study.

Previous studies [14, 34] have confirmed that anxiety and depression are more common in patients with FGIDs than in the healthy population, particularly in patients with IBS, as confirmed here. Anxiety, depression, and life event stress were more prevalent in patients with IBS than in healthy controls. However, whether patients IBS and SIBO have more severe psychological disorders is unknown. Our study showed similar levels of anxiety and depression, including life event stress experience. A possible explanation may be that the symptoms of SIBO are similar to those of IBS, such as bloating, diarrhoea, abdominal pain, and malabsorption. Thus, SIBO does not further aggravate the psychological status in patients with IBS. This finding was consistent with Grover et al. [35], who reported no difference in psychological distress between SIBO-positive and SIBO-negative patients with IBS. Thus, it seems unlikely that psychological distress mediates the association between SIBO and bowel symptoms. However, one study found an increase in the number of positive breath tests in patients with fibromyalgia, suggesting an association between psychological distress factors, such as somatisation, and SIBO [36]. Another study demonstrated that state anxiety is related to SIBO [37]. No study has investigated psychological distress as a moderating variable in patients with SIBO.

Many studies have demonstrated that some patients with IBS display persistent signs of low-grade mucosal inflammation, with activated T lymphocytes, B lymphocytes, and mast cells and increased expression of proinflammatory cytokines such as TNF-*α* and IL-6 [38–41]. We found that serum IL-10 level was significantly lower in SIBO-positive than SIBO-negative patients with IBS, whereas the TNF-*α*, IL-6, and IL-8 levels were similar in both groups. IL-10 is an anti-inflammatory cytokine produced by T cells, B cells, and monocytes and inhibits synthesis of other cytokines. Thus, a decrease in the IL-10 level could predispose a patient to increased mucosal cytokine production during infections or other mucosal insults [42]. Intestinal inflammation and activation of the immune response can cause a cytokine imbalance. Experimental data suggest that inflammation, even if mild, can lead to persistent changes in gastrointestinal nerve and smooth muscle function, resulting in colonic dysmotility, hypersensitivity, and dysfunction [43]. It has also been reported from animal experiments that low-grade gut inflammation alters gastrointestinal tract motor function [20, 44]. Gut motility abnormalities can further predispose to bacterial overgrowth. Many studies have reported that a delayed OCTT is associated with SIBO in patients with diabetes mellitus, ulcerative colitis, and several other diseases [45–47].

This is the first report of lower peripheral serum IL-10 levels in SIBO-positive than SIBO-negative patients with IBS. Many studies have reported that IL-10 expression is lower in patients with IBS than in healthy controls [48–50], but studies on changes in peripheral cytokines in patients with IBS with or without SIBO are rare. Riordan et al. [51] investigated patients with SIBO by culturing proximal small intestinal luminal secretions and measuring luminal interferon-*γ*, IL-6, and TNF-*α* concentrations. They found increased mucosal production of IL-6 in SIBO-positive subjects. The subjects they recruited were patients with pure SIBO, not patients with IBS, and cytokines were assessed from intestinal luminal secretions, which may reflect cytokine levels more accurately than peripheral blood. In animal experiments, German et al. [52] examined the role of cytokines in the immunopathogenesis of SIBO in German shepherd dogs. Duodenal mucosal biopsies were taken, and mRNA expression of various cytokines was determined by semiquantitative reverse transcriptase polymerase chain reaction. IL-2, IL-5, IL-12p40, TNF-*α*, and transforming growth factor-*β*1 mRNA levels in SIBO-positive dogs were significantly greater than those in SIBO-negative dogs.

This study had some limitations. First, relatively small numbers of patients and controls were recruited. Second, we did not test cytokine profiles in the healthy controls. Third, we assessed cytokine profiles in peripheral blood rather than in mucosa or tissue. Measuring cytokine levels in the intestine or colon may be more appropriate. Future studies will assess activation of the immune response in the mucosa of SIBO-positive patients with IBS and healthy controls.

In summary, we conclude that SIBO is unlikely to be associated with older age, sex, alcohol consumption, or psychological disorders. Our results suggest that lower serum production of IL-10 occurs in SIBO-positive than SIBO-negative patients with IBS. Future studies will assess low-grade inflammation and immune activation status by determining the imbalance of cytokines and immune cell changes from intestinal biopsies in patients with IBS and SIBO. Such data will be valuable to develop a better understanding of the role of SIBO in the pathogenesis of IBS.

## Figures and Tables

**Table 1:** Baseline demographic factors of the patients and controls.

	Patients with IBS	Healthy controls	*p* value
	(*n* = 89)	(*n* = 13)	
Age in years, mean ± SD	45.7 ± 12.9	43.3 ± 8.6	0.595
Sex, male/female	47/42	9/4	0.266
BMI, kg/m^2^	22.1 ± 5.8	24.5 ± 2.9	0.245
Anxiety, mean ± SD	5.17 ± 3.16	2.77 ± 1.64	0.009
Depression, mean ± SD	5.52 ± 3.30	3.54 ± 2.50	0.041
LES	40.0 (11.0–75.0)	8.0 (0.0–35.0)	<0.001

**Table 2:** Baseline comparison of the small intestinal bacterial overgrowth- (SIBO-) positive and negative patients.

	SIBO (+) (*n* = 35)	SIBO (−) (*n* = 54)	*p* value
Age in years, mean ± SD	43.5 ± 12.5	47.2 ± 13.1	0.187
Sex, male/female	21/14	26/28	0.274
BMI, kg/m^2^	23.2 ± 7.7	21.4 ± 4.0	0.139
IBS-D	28	35	0.124
IBS-C	1	2	1.000
IBS-M	6	15	0.248
IBS-U	0	2	0.517

**Table 3:** General characteristics of the small intestinal bacterial overgrowth- (SIBO-) positive and negative patients with irritable bowel syndrome.

Variables	Levels	SIBO (+) (*n* = 35)	SIBO (−) (*n* = 54)	*p* value
Age, years	Mean ± SD	43.5 ± 12.5	47.2 ± 13.1	0.187

Sex, *n* (%)	Male	21 (60.0%)	26 (48.1%)	
	Female	14 (40.0%)	28 (51.9%)	0.274

BMI, kg/m^2^	Mean ± SD	23.2 ± 7.7	21.4 ± 4.0	0.139

Marital status, *n* (%)	Married	29 (82.9%)	52 (96.3%)	
	Single	6 (17.1%)	2 (3.7%)	0.030

Education, *n* (%)	≤Primary	8 (22.9%)	11 (20.4%)	
	Middle school	10 (28.6%)	19 (35.2%)	
	High school	9 (25.7%)	15 (27.8%)	0.848
	≥College	8 (22.9%)	9 (16.7%)	

Average family income, *n* (%)	<1000	3 (8.6%)	2 (3.7%)	
	≥1000	18 (51.4%)	35 (64.8%)	0.470
	≥5000	10 (28.6%)	10 (18.5%)	
	≥10000	4 (11.4%)	7 (13.0%)	

Job, *n* (%)	Office work	4 (11.4%)	6 (11.1%)	
	Physical work	22 (62.9%)	36 (66.7%)	0.924
	Housework	9 (25.7%)	12 (22.2%)	

Cigarette smoking, *n* (%)	Never	24 (68.6%)	44 (81.5%)	
	≥1	7 (20.0%)	7 (13.0%)	0.520
	≥10	2 (5.7%)	1 (1.9%)	
	≥20	2 (5.7%)	2 (3.7%)	

Alcohol drinking, *n* (%)	Never	16 (45.7%)	25 (46.3%)	
	Sometimes	17 (48.6%)	26 (26.1%)	0.840
	Often	0 (0.0%)	1 (1.9%)	
	Always	2 (5.7%)	2 (3.7%)	

Medical history, *n* (%)	No	27 (77.1%)	39 (72.2%)	0.604
	Yes	8 (22.9%)	15 (27.8%)	

**Table 4:** Hospital anxiety and depression scale and life event stress scores in patients with irritable bowel syndrome with and without small intestinal bacterial overgrowth (SIBO).

	SIBO (+) (*n* = 35)	SIBO (−) (*n* = 54)	Healthy controls (*n* = 13)
Anxiety (mean ± SD)	5.37 ± 3.61^*∗*^	5.04 ± 2.85^*∗*^	2.77 ± 1.64
Depression (mean ± SD)	5.86 ± 3.90^*∗*^	5.30 ± 2.87^*∗*^	3.54 ± 2.50
Life event stress	42.0 (11.0–99.0)^*∗*^	39.50 (0.0–69.25)^*∗*^	8.0 (0.0–35.0)

^*∗*^ *p* < 0.05 compared with healthy controls.

**Table 5:** Serum cytokine levels in small intestinal bacterial overgrowth- (SIBO-) positive and negative patients (pg/mL).

	SIBO (+) (*n* = 35)	SIBO (–) (*n* = 54)	*p* value
TNF-*α*	9.77 (5.16–15.24)	9.41 (6.09–13.49)	0.938
IL-6	6.84 (6.69–7.07)	6.76 (6.65–7.13)	0.678
IL-8	17.67 (5.30–36.19)	12.49 (5.94–48.96)	0.911
IL-10	12.92 (11.40–14.85)	14.03 (12.66–16.33)	0.026

## Additional Points

Small intestinal bacterial overgrowth (SIBO) has been implicated in the pathogenesis of irritable bowel syndrome (IBS), although the issue remains controversial. Psychosocial factors and low-grade colonic mucosal immune activation have been suggested to play important roles in the pathophysiology of IBS. Our results suggest that lower serum production of IL-10 occurs in SIBO-positive than SIBO-negative patients with IBS.
